# A Delayed Postoperative C5 Palsy due to Spinal Cord Lesion: A Typical Clinical Presentation but Unusual Imaging Findings

**DOI:** 10.1155/2016/7810734

**Published:** 2016-12-18

**Authors:** Nobuaki Tadokoro, Yusuke Kasai, Katsuhito Kiyasu, Motohiro Kawasaki, Ryuichi Takemasa, Masahiko Ikeuchi

**Affiliations:** Department of Orthopaedic Surgery, Kochi Medical School, Kochi University, Kochi, Japan

## Abstract

Postoperative C5 palsy (C5 palsy) is a troublesome complication after cervical spine surgery and its etiology is still unclear. We experienced a case of C5 palsy after anterior decompression with fusion for cervical ossification of posterior longitudinal ligament with the typical clinical presentation of left deltoid and bicep weakness and left-arm pain without deterioration of myelopathy symptoms, albeit with the unusual imaging findings not shown preoperatively of a swelling in the spinal cord, and intramedullary high intensity change on T2-weighed MRI. The additional posterior surgery was carried out to decompress the swollen spinal cord. The abnormal findings disappear on MRI taken three weeks following the second surgery and the weakness improved fully within three months after the second surgery. This case report highlights the possibility of spinal cord lesion due to circulatory impairment as a cause of C5 palsy.

## 1. Introduction

Postoperative C5 palsy (C5 palsy) is defined as the deltoid/bicep muscle weakness without any deterioration of myelopathy symptoms after cervical spine surgery [1], which often appear several days after surgery. Although the nerve root tethering produced by spinal cord shifting after spinal cord decompressive surgery is now becoming a leading hypothesis [2–4], the cause of C5 palsy is still controversial and the spinal cord lesion is also hypothesized as the probable mechanism [5, 6].

We report a unilateral C5 palsy case, in which perioperative imaging studies provide evidence of spinal cord lesion.

## 2. Case Presentation

**Figure 1:**
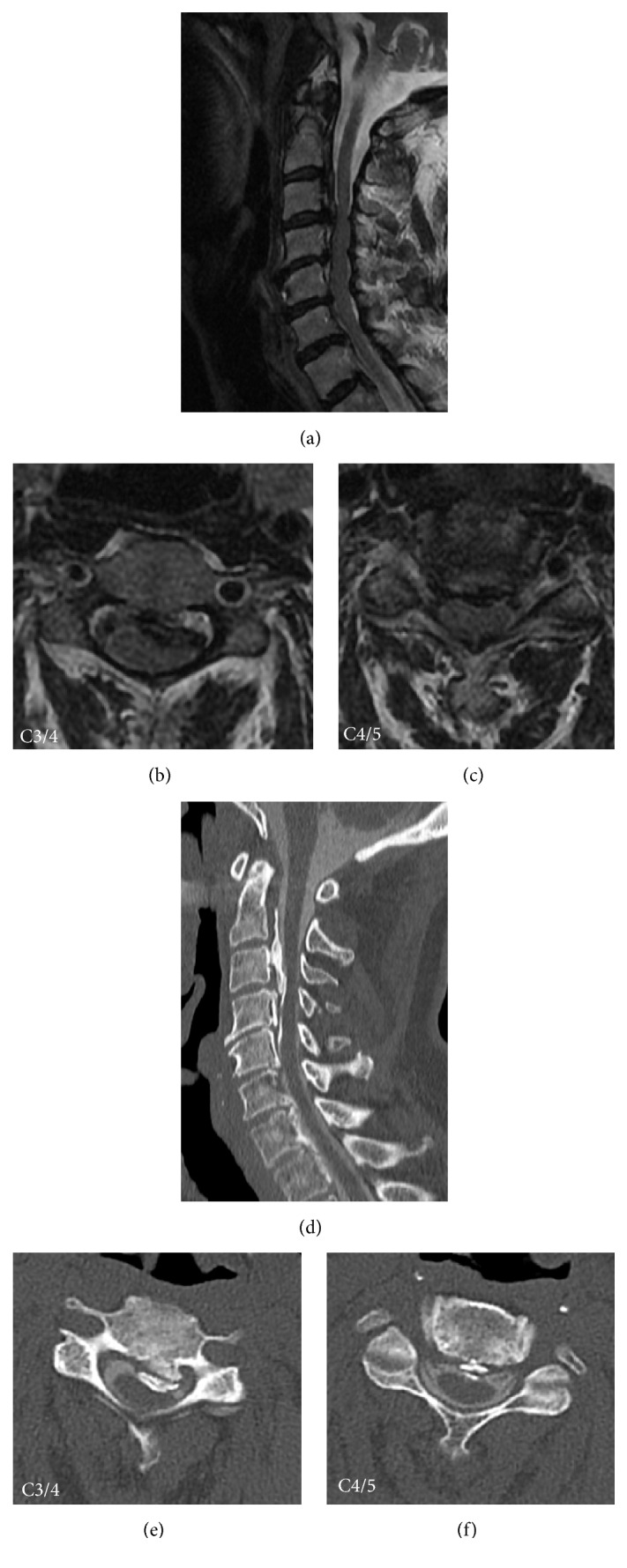
Preoperative images. Preoperative MRI (a, b, and c) and CT myelogram (d, e, and f) showed mixed-type OPLL compressing spinal cord, especially at C3-4 (OPLL canal occupying ratio: 50%). MRI showed no obvious cord signal change and cord enlargement (a, b, and c). The anterior-posterior diameters of right and left C4/5 foramen were 3.8 mm and 3.5 mm, respectively (f).

A 70-year-old female patient with cervical myelopathy presented at and was admitted to our hospital with aggravated quadriplegia due to ossification of posterior longitudinal ligament (OPLL). Her OPLL was mixed type from C2 vertebral body (VB) level down to C6 VB level, discontinuation at C3/4 with most cord compression, OPLL spinal canal occupancy ratio of 50%, and no C4/5 foraminal stenosis observed (Figure 1). Her clinical score, or cervical myelopathy Japanese Orthopaedic Association (JOA) score, was 9.5/17. There was no weakness of the deltoid and bicep muscles prior to surgery.

She underwent selective anterior cervical corpectomy of C4 and fusion with autoiliac bone grafting with plate fixation uneventfully. The intraoperative monitoring using transcranial electrical motor-evoked potentials with extremity muscle recordings including the deltoid and bicep muscles (MEP) and somatosensory-evoked potentials (SEP) showed no worsening of evoked potentials. Immediately after surgery, she experienced a favorable recovery of numbness in the extremities and no muscle weakness was observed.

**Figure 2:**
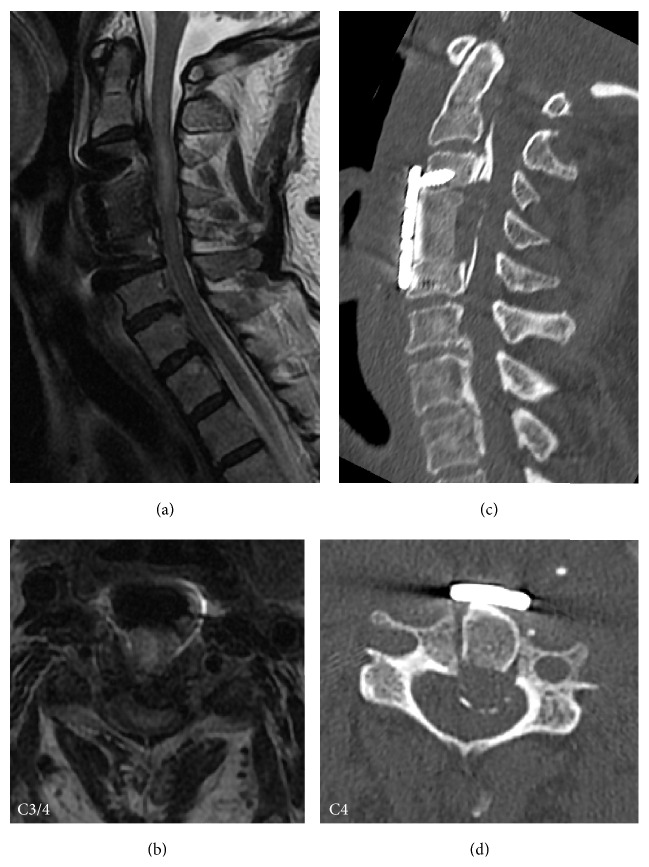
MRI and CT images taken at onset of C5 palsy. MRI (a, b) and CT imaging (c, d) obtained immediately after onset of C5 palsy showed the intramedullary T2 high signal change and spinal cord enlargement from C2 vertebral body level to C4 vertebral body level (a, b) without any graft dislodgement or implant malposition (c, d).

The left deltoid and bicep weakness of manual muscle testing (MMT) 1~2 and left-arm pain occurred on the second postoperative day, though her symptoms such as walk disturbance, difficulty of hand dexterity, and numbness of extremities maintained postoperative improvement after the onset of left-arm symptoms. Imaging studies on the same day of left-arm symptoms onset demonstrated T2 high intramedullary signal change and spinal cord enlargement from C2 VB level down to C4 VB level not evident prior to surgery on cervical MRI and no graft and implant malposition on CT images (Figure 2). Brain lesion was also ruled out by MRI and CT images. We diagnosed the patient with C5 palsy.

**Figure 3:**
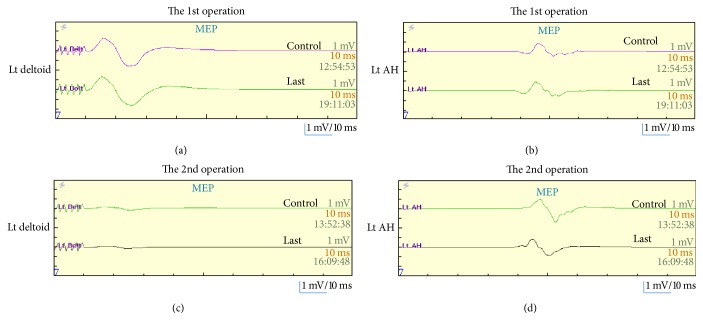
Intraoperative spinal cord monitoring. Although there was no worsening of left deltoid and left AH CMAPs during the 1st (a, b) and the 2nd (c, d), each operation was observed and low amplitude of left deltoid (c) and preserved amplitude of left AH CMAPs (d) in the 2nd operation compared to the 1st operation (a, b) were recorded, which means segmental C5 palsy occurred after the postoperative period of the 1st operation.

**Figure 4:**
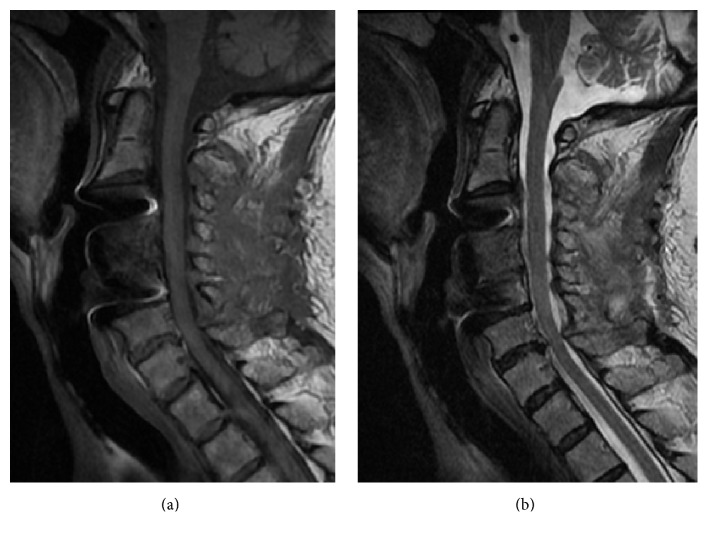
MRI taken three weeks after the second round of surgery. The spinal cord enlargement and intramedullary high intensity change disappear on T1 (a) and T2 (b) weighed MRI.

Although left-arm pain disappeared soon after administration of pregabalin, weakness of the deltoid and bicep muscles and T2 high intramedullary signal change and spinal cord enlargement remained unchanged. We recommended the additional C3–6 open-door laminoplasty with left C4/5 foraminotomy to this patient to decompress the spinal cord and left C5 root. She agreed to undergo a second round of surgery, which was performed ten days after the initial surgery. The C4/5 foraminal stenosis was not observed intraoperatively. The left deltoid and bicep muscles demonstrated reduced amplitude throughout the second round of surgery and the amplitude and latency of the rest of the muscles in MEP and SEP were the same compared with the first round of surgery in the intraoperative spinal cord monitoring (Figure 3). T2 high intramedullary signal change disappeared on MRI taken three weeks after the second round of surgery (Figure 4). Her weakness started to recover after the second round of surgery and had recovered fully three months later. Her JOA score at the time of C5 palsy full recovery was 13.5/17 (recovery rate: 53.3%).

## 3. Discussion

Imaging studies after C5 palsy rarely demonstrate obvious spinal cord lesion or nerve root lesion; however, T2 high intramedullary signal change and spinal cord enlargement occurred after the onset of C5 palsy in this patient, which suggests spinal cord edema due to the impairment of circulation and is clear evidence of spinal cord lesion.

The fact that the spinal cord lesion caused only the selective unilateral C5 palsy was unclear; the longitudinal spinal cord lesion including the C5 motor segment after the first surgery and the left-oriented OPLL could partly explain that due to the ischemia-reperfusion injury [7]. Even though the reperfusion cord injury after spinal cord decompression might be excluded because of the residual cord compression induced by the cranial OPLL, the circulatory aspect of neural damage could be assumed by the intramedullary signal change and cord enlargement after C5 palsy onset and the early resolution after the second round of surgery.

In regard to the C4/5 foraminal stenosis, the mean diameters of C4/5 foramen with C5 palsy were reported below 2.7 mm [2–4]; however, that of this patient was 3.5 mm. No left C4/5 foraminal stenosis in the intraoperative findings also supports the spinal cord lesion responsible for C5 palsy.

The intraoperative spinal cord monitoring showed no worsening during the first round of surgery, which meant no intraoperative neurological damage. The deltoid and biceps muscle amplitude reduction, albeit the other MEP and SEP preservation, in the second round of surgery corresponds to the clinical picture of delayed-onset segmental C5 palsy without pyramidal and sensory tract injury.

The prognosis of C5 palsy with conservative treatment is generally good [8] but sometimes poor [9]. The possibility of symptom aggravation due to cord swelling could not excluded completely and the effect of this atypical imaging findings for the functional recovery was not clear. So, we performed the additional decompressive surgery for cord swelling and possible C5 root lesion, though not evident in imaging studies, which possibly affected her clinical course beneficially from the postoperative early resolution of cord abnormalities and good recovery of weakness.

## 4. Conclusion

We reported a case of a unilateral delayed segmental C5 palsy after anterior cervical decompression (corpectomy) with fusion. The clinical picture was typical for C5 palsy, but MRI showed clear evidence of spinal cord lesion possibly due to circulatory impairment. We recommend MRI investigation at the onset of postoperative neurological complications to examine the pathology in detail.
